# Our Contemporaries

**Published:** 1914-10

**Authors:** 


### OUR CONTEMPORARIES

---

**The American Journal of Surgery** will begin with the October number, the publication of a regular 32-page supplement devoted to Anaesthesia, under the editorship of Dr. F. Hoeffler McMechan, of Cincinnati, with the following collaborators: Dr. James T. Gwathmey, New York; Dr. Willis D. Gatch, Indianapolis, Ind.; Dr. William Harper De Ford, Des Moines, Ia.; Dr. Charles K. Teter, Cleveland, O.; Dr. E. I. McKesson, Toledo, O.; Dr. Isabella C. Herb, Chicago, Ill.; and Yandel Henderson, of Yale University.

---

**The Southern Medical Journal** editorially advocates the recognition of the services of Surgeon-General Gorgas, by establishing a secretaryship of a national department of health, after retiring him from the Army with the rank of Major General. With every eulogy of Col. Gorgas that can be written, we heartily agree. We agree also with the personal recognition of his services by awarding him the rank of Major General and will go further by stating that, in our opinion, the Surgeon General of each of the medical corps should at least be eligible, regularly, to the same rank on account of special merit, if not as a matter of routine. So far as the establishment of a secretary of health in the cabinet in concerned, we hold to our formerly expressed view that if any such department is created, it should be by separating the Public Health Service from the Treasury Department as it has outgrown its position as a subordinate branch of this department. Owing to the established precedent that the secretaries are appointees of the President, for a short term, we hesitate to second the nomination of Col. Gorgas to such a position, under present conditions. We believe that certain branches of government deserve an independent position, immediately subordinate only to the President but that some of these, including the proposed department of health, are, essentially, separate from political opinion and should be administered by permanent officials, whereas others, as originally constituted, are appropriately representative of immediate public opinion and should have heads constituting the personal cabinet of the President and appointed by him for the term of his office. Some adjustment of this problem should be made before the proposed department of public health can well be established. It is always dangerous to allow public matters to be arranged to fit an individual case, owing to the shortness of human life and the desirability to build not for a few years but for many generations. The U. S. Public Health Service has grown from a small beginning as an auxiliary medical service to the revenue fleet and, apparently because no other service was immediately available, to care for the merchant marine. Its scope has gradually been widened until its organization under the Secretary of the Treasury is justified only historically. When the time comes for the establishment of a national department of health, all that is necessary is to swing this service from a subordinate to a co-ordinate position and to give it slight additional power and considerable extra appropriation to increase its personnel and equipment. To side-track the Public Health Service would be both an injustice to a body of men that have made good and an economic waste. Neither do we regard it as sound policy to place over the actual and potential heads of any service, who have earned their promotions step by step in the regular way, one from another service, however deserving and distinguished. General Gorgas has won the highest rank and position in the service to which he belongs. It would be appropriate to confer upon him still higher rank, but instead of retiring him, to continue him in charge of his own service, possibly by special act of congress, beyond the regular age for retirement. But we cannot regard it either as a real honor to him or a wise action, to make him a sort of super-Surgeon-General of another service.

